# Individual Prognosis Regarding Effectiveness of a Therapeutic Intervention Using Pre-Therapeutic “Kinesiology Muscle Test”

**DOI:** 10.1100/tsw.2007.257

**Published:** 2007-10-22

**Authors:** Ingrid Waxenegger, P. Christian Endler, Beatrix Wulkersdorfer, Heinz Spranger

**Affiliations:** Interuniversity College for Health and Development Graz, Castle of Seggau, Austria

**Keywords:** prior to treatment test, effectiveness, total cholesterol values, kinesiology muscle test

## Abstract

Since a therapy's full positive effect and possible adverse effects are individual and not predictable for every single patient, scientists have been searching for methods to predict optimal effects of a therapy. This pilot study investigated the applicability of the “kinesiology muscle test” as a prognostic tool regarding effectiveness in a defined therapeutic procedure. Each of 11 test persons with elevated total cholesterol values received a naturopathic drug supposed to lower cholesterol level on a daily basis for eight consecutive weeks. Prior to treatment the “kinesiology muscle test” was performed, where the patient's ability to maintain a flexed position in a selected joint was evaluated. The resistance created by the patient against the tester's pressure was monitored. Being in touch with healthful or unhealthful chemical substances may, according to the kinesiology literature, increase or decrease this resistance. For testing purposes, the drug was placed onto the patients' skin. The ability of the brachioradial muscle to resist the tester's pressure was determined on a subjective scale (0-100%). The Pearson product-moment correlation coefficient between four variables (total cholesterol value before therapy, total cholesterol value after therapy, difference of total cholesterol values before and after therapy, prior to treatment kinesiology testing) was chosen. A significant correlation between the difference of total cholesterol values before-after and the prior to treatment test was found, as well as a significant correlation between the total cholesterol values after therapy and the prior to treatment kinesiology test.

## INTRODUCTION

Over the last century the enormous progress in medicine is above all based on the use and availability of highly effective medication. Due to an extremely rapid development in pharmacological therapy the spectrum of treatment has become almost boundless. Nevertheless, application of an effective drug carries the risk of side effects[1]. Although biological substances have to go through highly demanding admission procedures, the degree of incidence of positive effects and possible adverse effects are individual and not predictable for every single patient. This well known issue can be explained by the fact that field trials in pharmacological research have to be geared to standards of statistical mean values within a defined population. Drug effect on each individual is, within a range, dependent on the patients' individual state, such as symptoms, typology, age and gender. Since under certain circumstances the effect of a substance may individually vary, each patient may show different tolerance regarding a special drug or therapeutic intervention. Even a proper indication for a special drug does not guarantee a positive effect in every single patient.

To predict optimal and avoid adverse effects, conventional medical diagnostic methods developed pre-therapeutic analysis; e.g. antibiotic-antibiogramm[2], chemosensitivity test[3] and pre-therapeutic analysis of pathogenic oncogenes in the therapy of monoclonal antibodies[4]. In many cases they have already turned out to be very useful and almost indispensable. However, the practical difficulties of using anamnestic tests can be pointed out by the example of antibiogramms. Since the time span to deliver an antibiogramm exceeds the length of the actual antibiosis, the use of a broad-spectrum antibiotic is indicated in order to prevent eventual aggravation of patients' condition, while the antibiogramm is in progress.

The aim of the study presented here was to determine the applicability of the "kinesiology muscle test" as a prognostic tool regarding effectiveness in defined therapeutic procedures. Historically "kinesiology" was founded in 1964/65 based on observations by George Goodheart, an American chiropractor[5]. This method originally includes diagnostic and therapeutic aspects. Over the years, kinesiology has established its own interdisciplinary methods including the "manual muscle test" as a functional testing method for determining and identifying dysfunctions. Furthermore, it aims at providing a tool for physicians to recommend an ideal treatment for each condition[6]. The following study is focusing on the diagnostic aspect. While performing the kinesiology muscle test, the tester is evaluating the patients' ability to maintain a flexed position in a selected joint. The resistance created by the patient against the tester's pressure or tension, is subjectively monitored by the tester. External conditions, such as being in touch with healthful or unhealthful chemical substances, may, according to kinesiology literature, increase or decrease this resistance. Results of empirical studies about kinesiology considering scientific criteria are controversial and therewith quite unclear. A study about food tolerance and intolerance by Pothman[7] was not able to show validity using a kinesiology method. Another study, in which it has been tried to determine whether the participants were in touch with a substance or part of a placebo group, was not able to point out the relevance of the muscle test[8]. On the other hand, a study comparing kinesiology assessments with physiological parameters (ECG) and lab findings of conventional food- and drug- tolerance tests was showing a correlation[9].

The purpose of the following pilot study was to determine, if the individual effectiveness of a defined therapeutic procedure was predictable using the kinesiology muscle test.

## METHODS

In particular, it has been investigated if the decrease in total cholesterol blood values was or was not predictable prior to the therapy with Red Yeast Rice (RYR). In this fermented rice, a fungus, Monascus pupureus, is found. This is supposed to have a decreasing effect on total cholesterol values.

This pilot study included 11 patients (n=11; 7 women and 4 men), between 26 and 78 years old, with elevated total cholesterol (TC) values of about 310 mg/dl in average. (Normal values should be < 200 mg/dl). All participants were provided by an Austrian, naturopathically orientated praxis (W. Surböck, Mariazell). Prior to treatment kinesiology muscle test was included in their routinely performed course of treatment. The actual RYR- therapy was planed and performed by the physician in agreement with the patients. Prior to treatment muscle the first author, providing each volunteer with detailed information about the test in advance, performed test. Cholesterol value of each patient was evaluated by local laboratory (Dostal, Vienna), before and after therapy. Each test person received 2 capsules of RYR-extract (500 mg each) on a daily basis for eight consecutive weeks.

Before starting therapy the kinesiology muscle test was performed using the brachioradial muscle as an indicator muscle. Therefore, the patient, set in a reclined position, had to hold his or her arm in a special testing position, while the tester was applying a slight, slowly increasing pressure. To get in touch with the test substance, 500 mg of RYR was placed onto the patients' skin, 2 cm above the bellybutton. An experienced tester was evaluating, whether the indicator muscle was able to resist the pressure or if and at which point the muscle was loosing its strength, assuming that the strength of muscle resistance would positively correlate with the expected effect of the test substance.

The ability of the muscle to resist the tester's force was determined in steps of ten on a subjective scale (0-100%). Each value was documented, without the physician or patient's knowledge. Results of the prior to treatment kinesiology tests were disclosed after completing therapy and blood analysis.

In order to evaluate RYR-therapy effectiveness total blood cholesterol values were determined.

## Statistical Analysis

The Pearson product-moment correlation coefficient between 4 variables was used to perform statistical analysis. The total blood cholesterol levels and the prior to treatment kinesiology test represented the four variables.

- total cholesterol value prior therapy
- total cholesterol value after therapy
- difference of total cholesterol values prior and after therapy
- prior to treatment testing of effectiveness

## RESULTS

Based on the results, predictability regarding the effectiveness of a therapeutic procedure (TC value) can be determined using the kinesiology muscle test. Figure 1 shows the mean values of the total cholesterol values.

Since a significant difference between the total cholesterol values before and after RYR-therapy was determined, evaluation of results regarding prior to treatment kinesiology test seemed reasonable.

Table 1 shows the correlation between the total cholesterol values and prior to treatment kinesiology testing.

A significant relation between the total cholesterol values after therapy and the first prior to treatment kinesiology test of effectiveness was determined ($p = 0,036$). The higher the predicted effect, the lower the total cholesterol values after therapy.

Furthermore, a significant correlation between difference of total cholesterol values prior-after and the prior to treatment test can be shown ($p = 0,016$).

The initial individual cholesterol values do not have significant influence on the predictability of the actual effectiveness.

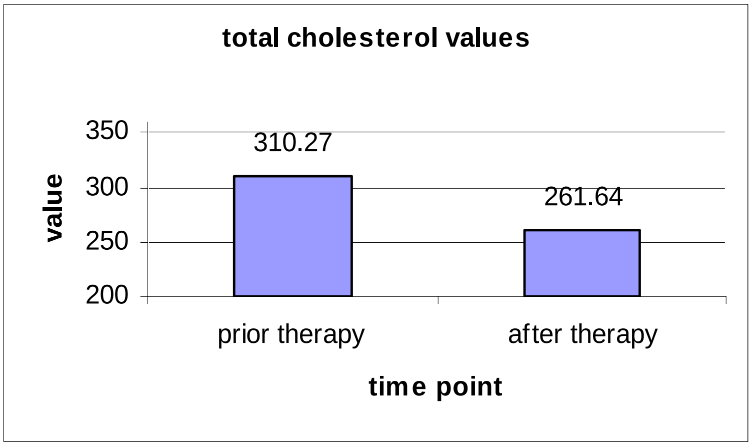

**Figure 1.** Total cholesterol values measured in mg/dl before and after RYR-therapy. Difference between the two measurements are significant (p = 0.003, 2-tail).

**Table 1.**
**Pearson correlation and 2-tail significance. TC = total cholesterol values**

| | | **TC before therapy** | **TC after therapy** | **TC-difference** | **Test prior to treatment** |
|---|---|---|---|---|---|
| **TC before therapy** | Corr. | 1 | ,496 | ,492 | ,065 |
| | Sign. | | ,121 | ,124 | ,850 |
| **TC after therapy** | Corr. | ,496 | 1 | -,512 | -,636(*) |
| | Sign. | ,121 | | ,108 | **,036** |
| **TC-difference** | Corr. | ,492 | -,512 | 1 | ,701(*) |
| | Sign. | ,124 | ,108 | | **,016** |
| **Test prior treatm.** | Corr. | ,065 | -,636(*) | ,701(*) | 1 |
| | Sign. | ,850 | **,036** | **,016** | |

## DISCUSSION

The aim of this pilot study was to determine the applicability of the kinesiology muscle test as a method of prognosis regarding effectiveness of the therapeutic intervention described above. Based on our results, intervention's effectiveness showed a significant statistical correlation with the prior to treatment assessment/estimation using the kinesiology muscle test.

Since each drug has a broad spectrum of effects and side effects, a detailed predictability of these effects on every single individual is nearly impossible. Thus, the kinesiology muscle test may represent a useful tool to optimize the election and the use of therapeutic, adjuvant and preventive interventions. Therefore, the tester has to fulfill certain requirements. Special training and qualification following medical guidelines is absolutely essential to provide professional tests. A medically indicated intervention should never be neglected on basis of pre-therapeutic tests.

For further and more detailed investigations a larger number of patients should be included and multiple independently performed kinesiology tests performed. A wider range of parameters should be considered: besides evaluating total cholesterol values, HDL- and LDL- fractions have to be included. An important aspect would be to standardize the performance of pre-therapeutic tests for conventional pharmaceuticals.

## References

1. Barry, E., Alvarez, J.A., Scully, R.E., Miller, T.L., and Lipshultz, S.E. (2007) Anthracycline-induced cardiotoxicity: course, pathophysiology, prevention and management. *Expert Opin Pharmacother.* **8**, 1039-1058.
2. Hindler, J.F. and Stelling, J. (2007) Analysis and presentation of cumulative antibiograms: a new consensus guideline from the Clinical and Laboratory Standards Institute. *Clin Infect Dis.* **15**, 867-873.
3. Kurbacher, C.M., Grecu, O.M., Stier, U., Gilster, T.J., Janat, M.M., Untch, M., Konecny, G., Bruckner, H.W., and Cree, I.A. (2003) ATP chemosensitivity testing in ovarian and breast cancer: early clinical trials. *Recent Results Cancer Res.* **161**, 221-230.
4. Sliwkowski, M.X., Lofgren, J.A., Lewis, G.D., Hotaling, T.E., Fendly, B.M., and Fox, J.A. (1999) Nonclinical studies addressing the mechanism of action of trastuzumab (Herceptin). *Semin Oncol.* **26**, 60-70.
5. Gin, R.H., Green, B.N. (1997) George Goodheart, Jr., D.C., and a history of applied kinesiology. *J Manipulative Physiol Ther.* **20**, 331-337.
6. Cuthbert, S.C. and Goodheart, G.J., Jr. (2007) On the reliability and validity of manual muscle testing: a literature review. *Chiropr Osteopat.* **15**, 4.
7. Pothman, R., Frankenberg, S.von, Hoicke, C., Weingarten, H., and Lüdtke, R. (2001). Evaluation der klinisch angewandten Kinesiologie bei Nahrungsmittel-Unverträglichkeiten im Kindesalter *Res Compl Med.,* **8**, 336-344.
8. Lüdtke, R., Kunz, B., Seeber, N., and Ring, J. (2001) Test-retest-reliability and validity of the Kinesiology muscle test. *Complementar Ther Med.* **9**, 141-5.
9. Omura, Y. (1981) New simple early diagnostic methods using Omura's "Bi-Digital O-Ring Dysfunction Localization Method" and acupuncture organ representation points, and their applications to the "drug & food compatibility test" for individual organs and to auricular diagnosis of internal organs - part I. *Acupunct Electrother Res.* **6**, 239-54.
